# Blastocyst quality and reproductive and perinatal outcomes: a multinational multicentre observational study

**DOI:** 10.1093/humrep/dead212

**Published:** 2023-10-24

**Authors:** Haowen Zou, James M Kemper, Elizabeth R Hammond, Fengqin Xu, Gensheng Liu, Lintao Xue, Xiaohong Bai, Hongqing Liao, Songguo Xue, Shuqin Zhao, Lan Xia, Jean Scott, Vincent Chapple, Masoud Afnan, Dean E Morbeck, Ben W J Mol, Yanhe Liu, Rui Wang

**Affiliations:** Department of Obstetrics and Gynaecology, Monash University, Clayton, Australia; Department of Obstetrics and Gynaecology, Monash University, Clayton, Australia; Monash Women’s, Monash Health, Clayton, Australia; Fertility Associates, Auckland, New Zealand; Department of Reproductive Medicine, Tianjin First Central Hospital, Tianjin, China; Centre for Reproductive Medicine, Tianjin Aiwei Hospital, Tianjin, China; Reproductive Medical and Genetic Center, People’s Hospital of Guangxi Zhuang Autonomous Region, Nanning, China; Department of Gynaecology and Obstetrics, Tianjin Medical University General Hospital, Tianjin, China; Department of Obstetrics and Gynaecology, The Second Affiliated Hospital of Hengyang Medical School, South China University, Hengyang, China; Center for Reproductive Medicine, Shanghai East Hospital, Shanghai, China; Center for Reproductive Medicine, Zaozhuang Marternal and Child Health Center, Zaozhuang, China; Reproductive Medical Center of Ruijin Hospital, School of Medicine, Shanghai Jiao Tong University, Shanghai, China; Fertility Solutions, Sunshine Coast, Australia; Fertility North, Joondalup, Australia; Qingdao United Family Hospital, Qingdao, China; Department of Obstetrics and Gynaecology, Monash University, Clayton, Australia; Fertility Associates, Auckland, New Zealand; Department of Obstetrics and Gynaecology, University of Auckland, Auckland, New Zealand; Department of Obstetrics and Gynaecology, Monash University, Clayton, Australia; Monash Women’s, Monash Health, Clayton, Australia; Aberdeen Centre for Women’s Health Research, School of Medicine, Medical Sciences and Nutrition, University of Aberdeen, Aberdeen, UK; Fertility North, Joondalup, Australia; School of Human Sciences, University of Western Australia, Crawley, Australia; School of Medical and Health Sciences, Edith Cowan University, Joondalup, Australia; Bond University, Robina, Australia; Department of Obstetrics and Gynaecology, Monash University, Clayton, Australia

**Keywords:** single blastocyst transfer, embryo quality, blastocyst grading, reproductive outcome, perinatal outcome

## Abstract

**STUDY QUESTION:**

Does the transfer of single low-grade blastocysts result in acceptable reproductive and perinatal outcomes compared to the transfer of single good-grade blastocysts?

**SUMMARY ANSWER:**

The transfer of single low-grade blastocysts resulted in a reduced live birth rate of around 30% (14% for very low-grade blastocysts) compared to 44% for single good-grade blastocysts, but does not lead to more adverse perinatal outcomes.

**WHAT IS KNOWN ALREADY:**

It is known that low-grade blastocysts can result in live births. However, the current studies are limited by relatively small sample sizes and single-centre designs. Furthermore, evidence on perinatal outcomes after transferring low-grade blastocysts is limited.

**STUDY DESIGN, SIZE, DURATION:**

We conducted a multi-centre, multi-national retrospective cohort study of 10 018 women undergoing 10 964 single blastocyst transfer cycles between 2009 and 2020 from 14 clinics across Australia, China, and New Zealand.

**PARTICIPANTS/MATERIALS, SETTING, METHODS:**

Blastocysts were graded individually based on assessment of the morphology and development of the inner cell mass (ICM) and trophectoderm (TE), and were grouped into three quality categories: good- (AB, AB, or BA), moderate- (BB), and low-grade (grade C for ICM or TE) blastocysts. CC blastocysts were individually grouped as very low-grade blastocysts. Logistic regression with generalized estimating equation was used to analyse the association between blastocyst quality and live birth as well as other reproductive outcomes. Binomial, multinomial logistic, or linear regression was used to investigate the association between blastocyst quality and perinatal outcomes. Odds ratio (OR), adjusted OR (aOR), adjusted regression coefficient, and their 95% CIs are presented. Statistical significance was set at *P *<* *0.05.

**MAIN RESULTS AND THE ROLE OF CHANCE:**

There were 4386 good-grade blastocysts, 3735 moderate-grade blastocysts, and 2843 low-grade blastocysts were included in the analysis, for which the live birth rates were 44.4%, 38.6%, and 30.2%, respectively. Compared to good-grade blastocysts, the live birth rate of low-grade blastocysts was significantly lower (aOR of 0.48 (0.41–0.55)). Very low-grade blastocysts were associated with an even lower live birth rate (aOR 0.30 (0.18–0.52)) and their absolute live birth rate was 13.7%. There were 4132 singleton live births included in the analysis of perinatal outcomes. Compared with good-grade blastocysts, low-grade blastocysts had comparable preterm birth rates (<37 weeks, aOR 1.00 (0.65–1.54)), birthweight Z-scores (adjusted regression coefficient 0.02 (0.09–0.14)), and rates of very low birth weight (<1500 g, aOR 0.84 (0.22–3.25)), low birth weight (1500–2500 g, aOR 0.96 (0.56–1.65)), high birth weight (>4500 g, aOR 0.93 (0.37–2.32)), small for gestational age (aOR 1.63 (0.91–2.93)), and large for gestational age (aOR 1.28 (0.97–1.70)).

**LIMITATIONS, REASONS FOR CAUTION:**

Due to the nature of the retrospective design, residual confounding could not be excluded. In addition, the number of events for some perinatal outcomes was small. Between-operator and between-laboratory variations in blastocyst assessment were difficult to control.

**WIDER IMPLICATIONS OF THE FINDINGS:**

Patients undergoing IVF should be informed that low-grade blastocysts result in a lower live birth rate, however they do not increase the risk of adverse perinatal outcomes. Further research should focus on the criteria for embryos that should not be transferred and on the follow-up of long-term outcomes of offspring.

**STUDY FUNDING/COMPETING INTEREST(S):**

H.Z. is supported by a Monash Research Scholarship. B.W.J.M. is supported by a NHMRC Investigator grant (GNT1176437). R.W. is supported by an NHMRC Emerging Leadership Investigator grant (2009767). B.W.J.M. reports consultancy, travel support, and research funding from Merck. The other authors do not have competing interests to disclose.

**TRIAL REGISTRATION NUMBER:**

N/A.

## Introduction

Single embryo transfer (SET) is widely accepted as an effective method to reduce the risk of multiple pregnancies and perinatal mortality (McLernon *et al.*, 2010; Sullivan *et al.*, 2012). The proportion of SET cycles has increased over the last two decades, reaching over 90% in Australia, New Zealand, and some Nordic countries (Newman and Chambers, 2021; Wyns *et al.*, 2022). In the era of SET, the role of selecting the best embryo for transfer becomes critical to minimize the time to pregnancy, resulting in a surge of interest in embryo selection technologies including PGT-A and time-lapse videography (Gardner *et al.*, 2015).

On the other side of the spectrum, the clinical outcomes after low-grade blastocyst transfers seem understudied due to the lack of clinical data. Assessment of the morphological quality of a blastocyst includes the expansion of blastocoel, and the cell number and structural arrangement of the inner cell mass (ICM) and trophectoderm (TE) (Gardner and Schoolcraft, 1999).

While existing guidelines and consensus (Alpha Scientists in Reproductive Medicine and ESHRE Special Interest Group of Embryology, 2011) do not recommend discarding low-grade blastocysts (De los Santos *et al.*, 2016; Morbeck, 2017; Practice Committees of the American Society for Reproductive Medicine (ASRM) and the Society for Reproductive Biologists and Technologists (SRBT), 2022), some clinics across the world routinely discard low-grade blastocysts as a matter of policy (Rubio *et al.*, 2014; Comstock *et al.*, 2015; Munch *et al.*, 2015; Lai *et al.*, 2020).

Importantly, low-grade blastocysts still have the potential to result in live births (Licciardi *et al.*, 2015; Bouillon *et al.*, 2017; Arab *et al.*, 2021; Li *et al.*, 2022). These contribute to cumulative live births (CLBRs) per oocyte collection, which is considered the most important treatment outcome for couples undergoing IVF (Cornelisse *et al.*, 2022). However, a scoping review published in 2021 showed that the evidence regarding reproductive outcomes following transfer of low-grade blastocysts remains limited, due to their relatively small sample sizes (n = 10–440) (Kemper *et al.*, 2021). Since the review, a single-centre study presented reproductive outcomes for 1104 grade BB or lower blastocysts transfers, of which 204 had a grade <BB (Arab *et al.*, 2021). Evidence regarding perinatal outcomes following transfer of low-grade blastocysts is even more sparse.

Therefore, we performed a large multi-national multi-centre observational study to evaluate the potential of low-grade blastocysts to establish a live birth, and the impact of blastocyst quality on perinatal outcomes for couples undergoing single blastocyst transfer.

## Materials and methods

### Study design and participants

This study was a multi-centre, multi-national retrospective cohort study. The ethics approval was obtained from Monash University Human Research Ethics Committee (2022-35404-80920). We included women undergoing IVF with a single blastocyst transfer in an autologous fresh or frozen cycle from 14 clinics across three countries (1 from Australia, 8 from China, and 5 from New Zealand) between 2009 and March 2020. We excluded: (i) cycles in which a cleavage embryo was transferred, (ii) cycles in which two or more blastocysts were transferred, and (iii) cycles in which stage 1 or 2 blastocysts were transferred (Gardner and Schoolcraft, 1999).

### IVF procedures

Ovarian stimulation for fresh cycles was performed according to local protocols at each clinic. Luteal support was performed as per clinical-specific protocol. Oocyte collection, sperm preparation, insemination, and embryo culture were conducted as per routine practice, with slight variations in protocols between clinics. Fertilized oocytes were cultured to either cleavage or blastocyst stage for transfer and/or cryopreservation. All suitable blastocysts were vitrified 5-, 6-, or 7-day post-oocyte collection. Women undergoing a single blastocyst transfer, of a fresh or frozen cycle embryo, were included in this study. For frozen embryo transfer cycles, blastocysts were warmed on the day of transfer and the grading was determined by embryologists, prior to embryo transfer.

### Blastocyst grading system

The Gardner blastocysts grading system was used to assess degree of blastocoel expanding and ICM/TE morphology (Gardner and Schoolcraft, 1999). We used A, B, or C to represent the quality of ICM and TE separately. The combination of developmental stage and ICM and TE quality formed the grading of each blastocyst. We classified blastocysts with an A or B in both ICM and TE as good-grade blastocysts (AA, AB, or BA), those with a B in both ICM and TE as moderate-grade blastocysts (BB), and those with a C in either ICM or TE as low-grade blastocysts (AC, CA, BC, CB, CC). Very-low grade blastocysts referred to CC blastocysts only.

### Outcome measures

The reproductive outcomes of interest included live birth, clinical pregnancy, pregnancy loss, and multiple birth. The perinatal outcomes included gestational age and birth weight. Gestational age was presented as either preterm birth (PTB, defined as gestational weeks <37) or term birth. Birth weight was reported as: (i) birthweight Z-scores; (ii) either very low birth weight (VLBW, defined as birth weight <1500 g), low birth weight (LBW, defined as 1500 g  ≤ birth weight <2500 g), normal birth weight (NBW, defined as 2500 g ≤ birth weight <4500 g), or high birth weight (HBW, defined as birth weight >4500 g); and (iii) either small for gestational age (SGA, defined as birth weight <10% for gestational age), appropriate for gestational age (APA), or large for gestational age (LGA, defined as birth weight >90% for gestational age). The birthweight percentiles and birthweight Z-scores were calculated based on the standards reported in the INTERGROWTH-21st Project and SGA and LGA were subsequently categorized (Villar *et al.*, 2014).

### Statistical analyses

Continuous variables were presented as means with SDs and categorical variables were presented as numbers with percentages. All women undergoing single blastocyst transfers were included in the analysis of reproductive outcomes (rates of live birth, clinical pregnancy, pregnancy loss, and multiple births). These outcomes were reported per blastocyst grading (good, moderate, and low) group, ICM grading (A, B, and C), and TE grading (A, B, and C), respectively. The absolute rates for each outcome per cycle were reported. For low-grade blastocysts, outcomes for each subgroup (AC, CA, BC, CB, and CC) were also presented where possible. Good-grade blastocysts, grade A ICM, or grade A TE were set as the references in the regressions, respectively. To account for the cluster effects of multiple embryo transfer cycles of the same couple, logistic regression via generalized estimating equations (GEEs) were used to estimate the association between the quality of blastocysts and reproductive outcomes (Dodge *et al.*, 2020). Both unadjusted and adjusted odds ratios with their corresponding 95% CI were reported. The following potential confounding factors were included in the adjusted model: institutions, transfer method (fresh/frozen), female age, blastocyst developmental stage, and blastocyst age. The unit of analysis in GEE for all reproductive outcomes were women undergoing embryo transfer. For multiple births, we performed a sensitivity analysis by using live birth after embryo transfer as the unit of analysis.

Margins plots were used to illustrate the impact of age on the associations between blastocysts grading and live birth, where the predictive live birth rate with 95% CIs per blastocyst grading were visualized against female age. Absolute live birth rates per blastocyst grading group stratified by quintiles of age were also tabulated.

In the analysis of the association between blastocyst quality and perinatal outcomes, only women with singleton births were included, whilst women with multiple births were excluded. For perinatal outcomes, infant sex was included as an additional confounding factor in the adjusted analysis. Binomial logistic regression was performed for the analysis of PTB. Linear regression was used for the analysis of birthweight Z-scores. Multinomial logistic regression was conducted for the analysis of VLBW/LBW/NBW/HBW as well as SGA/AGA/LGA. In addition, reproductive and perinatal outcomes between Day 6 and Day 5 low-grade blastocysts were also compared. All the analyses were performed in Stata/SE 17.0 and *P *<* *0.05 was considered statistically significant.

## Results

We included 10 018 women undergoing 10 964 single blastocyst transfer cycles. Among these women, 946 had between two and five transfer cycles. There were 4386 good-grade blastocysts, 3735 moderate-grade blastocysts, and 2843 low-grade blastocysts.

The demographic and embryo characteristics are presented in Table 1. The mean female age was 33 years. The majority of single blastocyst transfer cycles were frozen cycles (72%, 81%, and 91% in good, moderate, and low grading groups, respectively). Overall, 2209 (20.2%) were fresh transfers and 8755 (79.9%) were frozen transfers.

**Table 1.** Demographic and embryologic characteristics of embryo transfer cycles.

Characteristics/blastocyst grades		Good	Moderate	Low
		(4386 cycles)	(3735 cycles)	(2843 cycles)
**Female age, mean (SD)**		33.3 (4.8)	32.9 (4.8)	33.1 (5.0)
**Transfer method**	**Fresh**	1233 (28.1%)	716 (19.2%)	260 (9.2%)
	**Frozen**	3153 (71.9%)	3019 (80.8%)	2583 (90.9%)
**No. of cycles**	**1**	4183 (95.4%)	3375 (90.4%)	2460 (86.5%)
	**2–5**	203 (4.6%)	360 (9.6%)	383 (13.5%)
**Blastocyst developmental stage**	**3**	441 (10.1%)	954 (25.5%)	926 (32.6%)
	**4**	2879 (65.6%)	2385 (63.9%)	1676 (59.0%)
	**5**	1009 (23.0%)	344 (9.2%)	185 (6.5%)
	**6**	57 (1.3%)	52 (1.4%)	56 (2.0%)
**Blastocyst age (days)**	**5**	3976 (90.7%)	3108 (83.2%)	1736 (61.1%)
	**6**	395 (9.0%)	604 (16.2%)	1078 (37.9%)
	**7**	15 (0.3%)	23 (0.6%)	29 (1.0%)

Data are presented in mean ± SD or N (%)

### Reproductive outcomes

The reproductive outcomes stratified by overall blastocyst grading, ICM, and TE groups are presented in Table 2.

**Table 2.** Association between blastocyst grading and reproductive outcomes.

Outcomes	Overall blastocyst grading			ICM grading			TE grading		
	Good (N = 4386)	Moderate (N = 3735)	Low (N = 2843)	A (N = 3719)	B (N = 6401)	C (N = 844)	A (N = 3007)	B (N = 5841)	C (N = 2116)
**Live birth**	1946 (44.4%)	1443 (38.6%)	858 (30.2%)	1667 (44.8%)	2384 (37.2%)	196 (23.2%)	1368 (45.5%)	2201 (37.7%)	678 (32.0%)
**Crude OR**	Reference	**0.79** (0.72–0.86)	**0.54** (0.49–0.60)	Reference	**0.73** (0.67–0.79)	**0.37** (0.31–0.44)	Reference	**0.72** (0.66–0.79)	**0.56** (0.50–0.63)
**Adjusted OR**	Reference	**0.70** (0.62–0.77)*	**0.48** (0.41–0.55)*	Reference	**0.79** (0.70–0.88)**	**0.40** (0.32–0.49)**	Reference	**0.75** (0.67–0.84)***	**0.58** (0.49–0.68)***
**Clinical pregnancy**	2268 (51.7%)	1816 (48.6%)	1149 (40.4%)	1949 (52.4%)	3010 (47.0%)	274 (32.5%)	1566 (52.1%)	2773 (47.5%)	894 (42.3%)
**Crude OR**	Reference	**0.88** (0.81–0.96)	**0.63** (0.58–0.70)	Reference	**0.81** (0.74–0.87)	**0.44** (0.37–0.51)	Reference	**0.83** (0.76–0.91)	**0.67** (0.60–0.75)
**Adjusted OR**	Reference	**0.71** (0.64–0.79)*	**0.48** (0.41–0.55)*	Reference	**0.77** (0.69–0.86)**	**0.40** (0.33–0.49)**	Reference	**0.78** (0.70–0.88)***	**0.59** (0.50–0.70)***
**Multiple birth**	19 (0.4%)	34 (0.9%)	15 (0.5%)	14 (0.4%)	53 (0.8%)	1 (0.1%)	15 (0.5%)	39 (0.7%)	14 (0.7%)
**Crude OR**	Reference	**2.11** (1.20–3.71)	1.22 (0.62–2.40)	Reference	**2.21** (1.22–3.99)	0.31 (0.04–2.39)	Reference	1.34 (0.74–2.44)	1.33 (0.64–2.76)
**Adjusted OR**	Reference	1.65 (0.81–3.38)*	1.15 (0.47–2.80)*	Reference	2.07 (0.98–4.39)**	0.40 (0.04–3.56)**	Reference	0.84 (0.43–1.65)***	0.74 (0.31–1.76)***
**Pregnancy loss**	322 (7.3%)	373 (10.0%)	291 (10.2%)	282 (7.6%)	626 (9.8%)	78 (9.2%)	198 (6.6%)	572 (9.8%)	216 (10.2%)
**Crude OR**	Reference	**1.40** (1.20–1.64)	**1.44** (1.22–1.70)	Reference	**1.32** (1.14–1.53)	1.24 (0.95–1.61)	Reference	**1.54** (1.30–1.82)	**1.61** (1.32–1.98)
**Adjusted OR**	Reference	1.04 (0.87–1.25)*	0.97 (0.76–1.22)*	Reference	0.90 (0.75–1.09)**	0.82 (0.59–1.14)**	Reference	1.14 (0.93–1.39)***	1.06 (0.81–1.38)***

* Adjusted for institute, female age, fresh/frozen transfer, blastocyst developmental stage, blastocyst age.

** Adjusted for institute, female age, fresh/frozen transfer, blastocyst developmental stage, blastocyst age, TE grade.

*** Adjusted for institute, female age, fresh/frozen transfer, blastocyst developmental stage, blastocyst age, ICM grade.

ICM, inner cell mass; TE, trophectoderm; OR, odds ratio. Results in bold are *P<*0.05. Crude OR is calculated without any confounders.

The live birth rates were 44.4%, 38.6%, and 30.2% in good, moderate, and low-grade blastocysts groups, respectively. Compared to the good blastocyst grade group, the low and moderate blastocyst grade groups were associated with a lower live birth rate (low vs good: adjusted odds ratio (aOR) 0.48, 95% CI 0.41–0.55; moderate vs good: aOR 0.70, 95% CI 0.62–0.77). Compared to blastocysts with grade A ICM, blastocysts with grade C and B ICM were associated with a lower live birth rate (C vs A: aOR 0.40, 95% CI 0.32–049; B vs A: aOR 0.79, 95% CI 0.70–0.88). Similar findings were observed for TE grading (C vs A: aOR 0.58, 95% CI 0.49–0.68; B vs A: aOR 0.75, 95% CI 0.67–0.84). The findings on clinical pregnancy rates were overall consistent with those on live birth rates (Table 2).

When breaking down the low grading groups based on both ICM and TE grading, the absolute live birth rates per cycle for the CC and CB groups were 13.7% and 24.6%, respectively, whereas those for AC, CA and BC groups were similar (33%). CC, CB, and BC blastocysts were also associated with a significant lower chance of reaching live birth (CC vs good: aOR 0.30, 95% CI 0.18–0.52; CB vs good: aOR 0.35, 95% CI 0.28–0.43; BC vs good: aOR 0.53, 95% CI 0.46–0.63), compared to good grade blastocysts (Supplementary Table S1). It was not possible to perform regressions for CA and AC blastocysts due to the small numbers.

**Figure 1.**
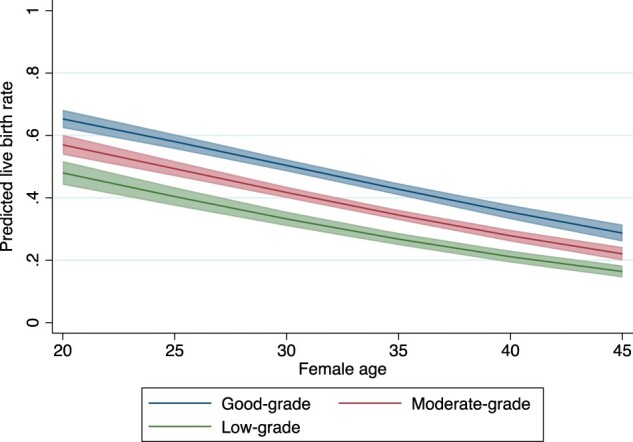
**Association of blastocyst grading and predicted live birth rate for women at different ages**.

Figure 1 shows the lower predicted live birth rate in women of more advanced ages across all blastocyst grading groups. For instance, women aged 25 undergoing a low-grade blastocyst transfer have a predicted live birth rate of 40% (95% CI 37–43%), whereas the predicted live birth rate for women aged 35 and undergoing a low-grade blastocyst transfer was 21% (95% CI 19–23%, Fig. 1). The absolute live birth rates per blastocyst grading group in five age quintiles are tabulated in Supplementary Table S2. While the live birth rate was lower in women with advanced age as well as women with lower blastocyst grade, it remained over 15% for women in the last quintile age group with a low-grade blastocyst.

There were 19 (0.4%), 34 (0.9%), and 15 (0.5%) twin births in the good, moderate, and low grading blastocysts groups, respectively. There were no triplets or high-order multiple pregnancies. There were no statistically significant differences in twin births rates between different blastocyst grading groups (low vs good; aOR 1.15, 95% CI 0.47–2.80), ICM grading groups (C vs A; aOR 0.40, 95% CI 0.04–3.56), or TE group (C vs A; aOR 0.74, 95% CI 0.31–1.76). In a sensitivity analysis using live birth as the unit of analysis, blastocysts with grade B ICM were associated with a higher chance of twin live birth compared to those with grade A ICM (aOR 2.41, 95% CI 1.08–5.38) (Supplementary Table S3). No statistically significant differences were observed in other comparisons in this sensitivity analysis.

Regarding pregnancy loss, there were no statistically significant differences between low-grade blastocysts and good-grade blastocyst (aOR 0.97, 95% CI 0.76–1.22), or between moderate-grade blastocysts and good-grade blastocysts (aOR 1.04, 95% CI 0.87–1.25). In either the ICM or the TE grading groups, compared to grade A blastocysts, grade B and C blastocysts were not associated with higher pregnancy loss rates (Table 2). When breaking down the low-grade group, no statistically significant differences in rates of pregnancy loss were observed between CC (aOR 0.69, 95% CI 0.21–2.23), BC (aOR 1.01, 95% CI 0.78–1.30), or CB (aOR 0.99, 95% CI 0.71–1.37) blastocysts and good-grade blastocysts (Supplementary Table S1).

### Perinatal outcomes

There were 4132 singleton live births after single blastocyst transfer. There was no significant difference between low and good-grade blastocysts in the PTB rate (8.1% vs 6.4%, aOR 1.00, 95% CI 0.65–1.54), birthweight Z-score (adjusted regression coefficient 0.02, 95% CI −0.09 to 0.14), or rates of VLBW (0.6% vs 0.9%, aOR 0.84, 95% CI 0.22–3.25), LBW (4.6% vs 4.6%, aOR 0.96, 95% CI 0.56–1.65), HBW (1.9% vs 1.6%, aOR 0.93, 95% CI 0.37–2.32), SGA (3.2% vs 6.9%, aOR 1.63, 95% CI 0.91–2.93), or LGA (26.9% vs 17.5%, aOR 1.28, 95% CI 0.97–1.70) (Table 3).

**Table 3.** Association between blastocyst quality and perinatal outcomes.

Outcomes	Overall blastocyst grading			ICM grading			TE grading		
	Good (N = 1893)	Moderate (N = 1398)	Low (N = 841)	A (N = 1626)	B (N = 2311)	C (N = 195)	A (N = 1336)	B (N = 2134)	C (N = 662)
**Preterm birth**	122 (6.4%)	127 (9.1%)	68 (8.1%)	106 (6.5%)	195 (8.4%)	16 (8.2%)	83 (6.2%)	182 (8.5%)	52 (7.9%)
**Crude OR**	Reference	**1.45** (1.12–1.88)	1.28 (0.94–1.74)	Reference	**1.32** (1.03–1.69)	1.28 (0.74–2.22)	Reference	**1.41** (1.08–1.84)	1.29 (0.90–1.84)
**Adjusted OR**	Reference	1.18 (0.87–1.62)*	1.00 (0.65–1.54)*	Reference	0.97 (0.70–1.35)**	0.88 (0.46–1.69)**	Reference	1.15 (0.82–1.59)***	0.94 (0.59–1.52)***
**Birthweight Z-score**, **mean (SD)**	0.4 (1.1)	0.6 (1.0)	0.6 (1.0)	0.3 (1.1)	0.6 (1.0)	0.7 (1.0)	0.3 (1.1)	0.5 (1.0)	0.6 (1.0)
**Coefficient (crude)**	Reference	**0.21** (0.14–0.28))	**0.29** (0.21–0.38))	Reference	**0.23** (0.17–0.30)	**0.38** (0.23–0.53)	Reference	**0.20** (0.13–0.27)	**0.29** (0.19–0.38)
**Coefficient (adjusted)**	Reference	0.04 (−0.05 to 0.12))*	0.02 (−0.09 to 0.14)*	Reference	0.05 (−0.04 to 0.14)**	0.12 (−0.06 to 0.30)**	Reference	−0.01 (−0.09 to 0.08)***	−0.04 (−0.17 to 0.09)***
**Birthweight (VLBW/LBW/NBW/HBW)**									
**NBW**	Reference	Reference	Reference	Reference	Reference	Reference	Reference	Reference	Reference
**VLBW**	17 (0.9%)	12 (0.9%)	5 (0.6%)	17 (1.1%)	16 (0.7%)	1 (0.5%)	13 (1.0)	17 (0.8%)	4 (0.6%)
**Crude OR**	Reference	0.96 (0.46–2.02)	0.66 (0.24–1.80)	Reference	0.66 (0.33–1.31)	0.48 (0.06–3.62)	Reference	0.82 (0.40–1.70)	0.62 (0.20–1.92)
**Adjusted OR**	Reference	1.20 (0.46–3.15)*	0.84 (0.22–3.25)*	Reference	0.60 (0.22–1.62)**	0.52 (0.05–5.02)**	Reference	1.38 (0.55–3.46)***	0.99 (0.22–4.39)***
**LBW**	87 (4.6%)	74 (5.3%)	39 (4.6%)	77 (4.7%)	115 (5.0%)	8 (4.1%)	55 (4.1%)	114 (5.3%)	31 (4.7%)
**Crude OR**	Reference	1.16 (0.84–1.59)	1.01 (0.69–1.49)	Reference	1.05 (0.78–1.41)	0.85 (0.40–1.78)	Reference	1.30 (0.94–1.81)	1.14 (0.73–1.80)
**Adjusted OR**	Reference	1.14 (0.77–1.68) *	0.96 (0.56–1.65)*	Reference	0.84 (0.56–1.25)**	0.59 (0.25–1.39)**	Reference	**1.57** (1.05–2.36)***	1.36 (0.74–2.48)***
**HBW**	31 (1.6%)	19 (1.4%)	16 (1.9%)	27 (1.7%)	38 (1.6%)	1 (0.5%)	25 (1.9%)	26 (1.2%)	15 (2.3%)
**Crude OR**	Reference	0.83 (0.47–1.48)	1.16 (0.63–2.14)	Reference	0.99 (0.60–1.63)	0.30 (0.04–2.23)	Reference	0.65 (0.38–1.14)	1.22 (0.64–2.33)
**Adjusted OR**	Reference	0.72 (0.35–1.47) *	0.93 (0.37–2.32)*	Reference	0.76 (0.36–1.58)**	0.18 (0.02–1.53)**	Reference	0.72 (0.36–1.46)***	1.27 (0.48–3.34)***
**Birthweight (SGA/AGA/LGA)**									
**AGA**	Reference	Reference	Reference	Reference	Reference	Reference	Reference	Reference	Reference
**SGA**	130 (6.9%)	54 (3.9%)	27 (3.2%)	118 (7.3%)	89 (3.9%)	4 (2.1%)	94 (7.0%)	94 (4.4%)	23 (3.5%)
**Crude OR**	Reference	**0.58** (0.42–0.81)	**0.51** (0.33–0.77)	Reference	**0.56** (0.42–0.74)	**0.31** (0.11–0.86)	Reference	**0.64** (0.48–0.87)	**0.53** (0.33–0.84)
**Adjusted OR**	Reference	1.12 (0.77–1.64)*	1.63 (0.91–2.93)*	Reference	0.94 (0.65–1.36)**	0.83 (0.28–2.47)**	Reference	1.23 (0.86–1.76)***	1.83 (0.97–3.45)***
**LGA**	331 (17.5%)	323 (23.1%)	226 (26.9%)	275 (16.9%)	547 (23.7%)	58 (29.7%)	233 (17.4%)	475 (22.3%)	172 (26.0%)
**Crude OR**	Reference	**1.37** (1.15–1.63)	**1.66** (1.37–2.02)	Reference	**1.46** (1.25–1.72)	**1.96** (1.40–2.73)	Reference	**1.31** (1.10–1.57)	**1.59** (1.27–2.00)
**Adjusted OR**	Reference	1.17 (0.95–1.45)*	1.28 (0.97–1.70)*	Reference	1.20 (0.96–1.49)**	**1.51** (1.00–2.26)**	Reference	1.00 (0.80–1.24)***	1.04 (0.77–1.42)***

* Adjusted for institute, female age, fresh/frozen transfer, blastocyst developmental stage, blastocyst age, infant gender.

** Adjusted for institute, female age, fresh/frozen transfer, blastocyst developmental stage, blastocyst age, infant gender, TE grade.

*** Adjusted for institute, female age, fresh/frozen transfer, blastocyst developmental stage, blastocyst age, infant gender, ICM grade.

ICM, inner cell mass; TE, trophectoderm; OR, odds ratio; VLBW: very low birth rate; LBW: low birth rate; NBW: normal birth weight; HBW: high birth weight; SGA: small for gestational age; AGA: appropriate for gestational age; LGA: large for gestational age. Results in bold are *P*<0.05. Crude OR is calculated without any confounders.

Similarly, we did not find any differences between grades C and A ICM or TE comparisons in these perinatal outcomes, except for LGA, where blastocysts with grade C ICM were associated with a borderline higher chance of LGA compared to those with grade A ICM blastocysts (aOR 1.51, 95% CI 1.00–2.26).

When comparing different low-grade subgroups to the good grade groups, the findings were consistent (Supplementary Table S4). It was not possible to perform regressions for the CC, CA, and AC groups due to the small numbers. When comparing Day 6 versus Day 5 low-grade blastocysts, we did not observe significant differences in perinatal outcomes (Supplementary Table S5).

## Discussion

### Summary of key findings

In this study, we found that compared to good-grade blastocysts (AA, AB, or BA), low-grade blastocysts (C in ICM or TE) were associated with a significantly lower, but still reasonable live birth rate of approximately 30% per transfer. Even the very low-grade blastocysts (CC) had a live birth rate of 14%. The findings were consistent in the analysis of the ICM and TE grading groups, respectively. With respect to age, even for women with advanced age, transferring a low-grade blastocyst still results in a live birth rate of over 15%. There were no differences between different blastocyst, ICM, or TE gradings in relation to pregnancy losses or perinatal outcomes (PTB, VLBW, LBW, HBW, SGA, or LGA).

### Strengths and limitations

By collaborating with 14 IVF clinics across three countries, we had a large sample size consisting of 10 018 women in the analysis of reproductive outcomes and 4132 singleton live births included in the analysis of perinatal outcomes. Furthermore, the population of interest was restricted to single blastocyst transfer cycles to remove the challenge of traceability of individual embryos and pregnancy outcomes. In addition, we prespecified a minimal set of confounding factors in the adjusted regression model to control bias due to confounding. Finally, we took the clustering effect of multiple cycles from the same couple into consideration by using GEE.

There are also limitations in this study. Firstly, due to the nature of the retrospective design, residual confounding cannot by avoided. Secondly, due to the long duration of the data collected (2009–2020) and the differences across multiple clinical registries, we were not able to collect additional patient characteristics across all centres, including duration of infertility, causes of infertility and other ovarian reserve, semen, and stimulation parameters. Thirdly, the sizes of some low-grade subgroups (AC and CC for reproductive outcomes; AC, CA, and CC for perinatal outcomes) were small and absolute numbers of some perinatal outcomes in the low grade/grade C ICM/TE groups (e.g. VLBW, SGA) were small. Lastly, the inter- and intra-operator variation in blastocyst grading is a known limitation in routine practice (Storr *et al.*, 2017).

### Interpretation and implications

Previous studies have shown that embryos with a higher grade or better quality can result in a significantly higher clinical pregnancy and live birth rates (Oron *et al.*, 2014; Bouillon *et al.*, 2017). Our study confirmed these findings. We would like to emphasize that we only included blastocyst transfer cycles in our study, which shows a higher live birth rate than registries including both blastocyst and cleavage transfers. In addition, cycles with no transferable embryos were not included. According to the 2019 SART report, when limited to SET, Day 5/6 transfer, and non-PGT cycles, the live birth rates per first and second/later transfer were 46.9% and 42.0%, respectively (SART, 2019). These are comparable to the live birth rates in our study. With respect to the live birth rate per transfer cycle of 30% in the low-grade blastocyst group, the figure is consistent with those in some studies, but not in other studies, as they varied from 15% to 60% (Wirleitner *et al.*, 2016; Li *et al.*, 2020; Awadalla *et al.*, 2021). The difference could be due to the differences in the definition of low-grade blastocysts (Li *et al.*, 2020) or the inclusion of PGT-A (Viñals Gonzalez *et al.*, 2019). Blastocysts with a C grade in ICM were associated with an even lower chance of live birth (25% for CB blastocysts and 14% for CC blastocysts), which indicated that the quality of the ICM might be a more determining factor than TE for live birth. When compared to blastocysts with a C grade in ICM, blastocysts with a C grade in TE were also significantly associated with a lower live birth rate, however the change was not as much as that for ICM C blastocysts. This is different from a previous single centre report, where blastocysts with a C grade in the ICM was not found to be associated with a lower live birth rate (Lai *et al.*, 2020). This is likely due to the smaller sample size and confounding factors that were not controlled for (e.g. the developmental stage of the blastocyst and the day of transfer) in the earlier study. Guo *et al.* (2020) reported that blastocysts with a C in either ICM or TE were independently correlated with live birth, which were consistent with our results. For women of advanced maternal age, the live birth rates were still high enough to justify the transfer of low-grade blastocysts.

With respect to multiple birth, ICM-B blastocysts were associated with a higher risk of multiple birth, compared with ICM-A blastocysts. This can be partly explained by the small number of twin births (68/10 964), with the majority being in the ICM B-grade blastocyst groups. This was not observed when comparing ICM-C and ICM-A blastocysts, as there was only one twin birth in the ICM-C group. A morphologically suboptimal ICM may be more vulnerable to a subsequent split due to structural disadvantages, such as loose intercellular connections. This needs to be further evaluated in future studies.

For PTB, our results indicated that suboptimal grade blastocysts were not associated with an increased risk of PTB, compared with good-grade blastocysts. The results from one single-centre study found no significant difference between blastocysts quality, either overall quality or ICM or TE grade, and PTB, which is consistent with our results (Hu *et al.*, 2021). For neonatal outcomes, including VLBW, LBW, and HBW, there were no significant association between blastocyst quality and perinatal outcomes. Similar findings were also observed in other studies (Oron *et al.*, 2014; Bouillon *et al.*, 2017; Li *et al.*, 2022). Our results of the analyses for SGA and LGA are consistent with Hu’s single-centre study, which reported that ICM-C blastocysts were associated with an increased risk of LGA and TE-C blastocysts were associated with an increased risk of SGA (Hu *et al.*, 2021). A recent study showed a higher rate of low-lying placentas, following the transfer of lower-quality embryos, but these placental lesions do not translate to adverse obstetric outcomes (Ganer Herman *et al.*, 2023) and merit further investigation in a large cohort.

Apart from the limited sample sizes in previous studies, another concern is that the significant heterogeneity in blastocyst grading between studies makes it difficult to compare the results and interpret the findings. Despite the Gardner blastocyst grading system being widely used (Bouillon *et al.*, 2017; Hu *et al.*, 2021), other grading systems, such as Society of Assisted Reproductive Technology (SART) criteria, could be adopted (Li *et al.*, 2022) and thus different thresholds for determining poor quality from good quality blastocysts exist (Oron *et al.*, 2014; Bouillon *et al.*, 2017). In future studies, a more standardized and accepted criteria should be developed. To achieve this, a numerical model incorporating three aspects of blastocyst morphological grades could be a start point, such as a recent study (Liu *et al.*, 2022), which calculated weightings of each aspect in predicting live birth likelihood.

Historically, low-grade blastocysts do not survive cryopreservation well in the slow freezing conditions, resulting in poor outcomes following embryo transfer. However, the development of vitrification has resulted in better viability and preservation of blastocysts, resulting in better reproductive outcomes. For couples who have no good or moderate-grade blastocysts for transfer, starting a new stimulation cycle may cause unnecessary delay in treatments, with extra cost for a new cycle and excessive psychological burden to the couples. Given that the CLBR is considered the most important outcome for women undergoing IVF (Cornelisse *et al.*, 2022), it would be important to evaluate the added value of transferring low-grade blastocysts in a complete IVF cycle, especially for women with multiple failed transfers with good quality blastocysts.

Blastocysts grading is essential in the selection of embryos and an indicator for the chance of implantation. However, evidence of the exploration of the association between low-grade blastocysts and live birth is scarce. Our study found that the live birth rate of CC blastocysts was 13.7%, which is close to the 16.7% live birth rate of CC blastocysts in another study (Li *et al.*, 2020). No statistically significant association was observed between the CC blastocysts and pregnancy loss. We therefore suggest that CC blastocysts can still result in a live birth and may be beneficial for patients with a limited number of good quality embryos, or repeated implantation failure. Moreover, the association between CC and perinatal outcomes need to be investigated in a larger cohort.

Patients undergoing IVF treatments should be informed that low-grade blastocysts result in lower, but reasonable live birth rates, without increasing the risk of adverse perinatal outcomes. Future research should focus on a criterion for embryos that should not be transferred. Moreover, other foetal outcomes, including placentation, preeclampsia, hypertensive disorders, and pregnancy complications, including haemorrhage and placenta previa should be investigated for more evidence regarding the use of low-grade blastocysts. While the neonatal outcomes following transfer of low-grade blastocysts are reassuring, long-term follow-up of childhood outcomes is also required.

## Supplementary Material

dead212_Supplementary_Table_S1

dead212_Supplementary_Table_S2

dead212_Supplementary_Table_S3

dead212_Supplementary_Table_S4

dead212_Supplementary_Table_S5

## Data Availability

The data underlying this article will be shared upon reasonable request to the corresponding author.
