# Supplementary material for: Blastocyst quality and reproductive and perinatal outcomes: a multinational multicentre observational study
Source: Hum Reprod. 2023 Oct 24;38(12):2391–9. doi: 10.1093/humrep/dead212 (PMC10694400; doi:10.1093/humrep/dead212)
Supplement: dead212_Supplementary_Table_S1 [file dead212_supplementary_table_s1.pdf]

OXFORD
human reproduction
Human Reproduction, 2023, 1–1
https://doi.org/10.1093/humrep/dead212
Supplementary Data

Supplementary Table S1. Association between different low-grade blastocysts and reproductive outcomes.

Outcomes	Good-grade (N = 4386)	AC (N = 27)	CA (N = 12)	BC (N = 1972)	CB (N = 715)	CC (N = 117)
**Live birth**	1946 (44.4%)	9 (33.3%)	4 (33.3%)	653 (33.1%)	176 (24.6%)	16 (13.7%)
**Crude OR**	Reference	/	/	**0.62** (0.56–0.69)	**0.41** (0.34–0.49)	**0.20** (0.12–0.34)
**Adjusted OR**	Reference	/	/	**0.53** (0.46–0.63)*	**0.35** (0.28–0.43)*	**0.30** (0.18–0.52)*
**Clinical pregnancy**	2268 (51.7%)	9 (33.3%)	4 (33.3%)	866 (43.9%)	251 (35.1%)	19 (16.2%)
**Crude OR**	Reference	/	/	**0.73** (0.66–0.81)	**0.51** (0.43–0.60)	**0.18** (0.11–0.30)
**Adjusted OR**	Reference	/	/	**0.54** (0.46–0.63)*	**0.37** (0.30–0.45)*	**0.32** (0.19–0.53)*
**Multiple birth**	19 (0.4%)	0	0	14 (0.7%)	1 (0.1%)	0
**Crude OR**	Reference	/	/	1.64 (0.82–3.28)	0.32 (0.04–2.41)	/
**Adjusted OR**	Reference	/	/	1.49 (0.59–3.72)*	0.36 (0.04–3.16)*	/
**Pregnancy loss**	322 (7.3%)	0	0	213 (10.8%)	75 (10.5%)	3 (2.6%)
**Crude OR**	Reference	/	/	**1.53** (1.27–1.84)	**1.48** (1.14–1.93)	0.33 (0.10–1.05)
**Adjusted OR**	Reference	/	/	1.01 (0.78–1.30)*	0.99 (0.71–1.37)*	0.69 (0.21–2.23)*

We did not perform regression analysis for AC or CA group due to small numbers.
* Adjusted for institute, female age, fresh/frozen transfer, blastocyst developmental stage, blastocyst age.
OR, odds ratio. Results in bold are *P*<0.05. Crude OR is calculated without any confounders. Bold values refer to statistically significant results.
