# Supplementary material for: Blastocyst quality and reproductive and perinatal outcomes: a multinational multicentre observational study
Source: Hum Reprod. 2023 Oct 24;38(12):2391–9. doi: 10.1093/humrep/dead212 (PMC10694400; doi:10.1093/humrep/dead212)
Supplement: dead212_Supplementary_Table_S2 [file dead212_supplementary_table_s2.pdf]

OXFORD
human reproduction
Human Reproduction, 2023, 1–1
https://doi.org/10.1093/humrep/dead212
Supplementary Data

Supplementary Table S2. Live birth rates in stratified age groups associated with the blastocysts grading.

Age (quintile)	Good (N = 4386)	Moderate (N = 3735)	Low (N = 2843)
20–29	542 (52.9%)	440 (46.9%)	267 (37.0%)
30–31	316 (49.3%)	283 (43.9%)	178 (39.4%)
32–34	460 (45.5%)	341 (42.6%)	194 (31.3%)
35–37	346 (40.9%)	224 (35.0%)	133 (27.8%)
≥38	282 (32.7%)	155 (21.8%)	86 (15.1%)
