# Supplementary material for: Blastocyst quality and reproductive and perinatal outcomes: a multinational multicentre observational study
Source: Hum Reprod. 2023 Oct 24;38(12):2391–9. doi: 10.1093/humrep/dead212 (PMC10694400; doi:10.1093/humrep/dead212)
Supplement: dead212_Supplementary_Table_S3 [file dead212_supplementary_table_s3.pdf]

OXFORD
human reproduction
Human Reproduction, 2023, 1–1
https://doi.org/10.1093/humrep/dead212
Supplementary Data

Supplementary Table S3. Sensitivity analysis on the association between blastocyst grading and multiple births.

	Overall blastocyst grading			ICM grading			TE grading		
**Outcomes**	**Good (N = 1946)**	**Moderate (N = 1443)**	**Low (N = 858)**	**A (N = 1667)**	**B (N = 2384)**	**C (N = 196)**	**A (N = 1368)**	**B (N = 2201)**	**C (N = 678)**
**Multiple birth**	19 (1.0%)	34 (2.4%)	15 (1.8%)	14 (0.8%)	53 (2.2%)	1 (0.5%)	15 (1.1%)	39 (1.8%)	14 (2.1%)
**Crude OR**	Reference	**2.45** (1.39–4.31)	1.80 (0.91–3.57)	Reference	**2.68** (1.48–4.85)	0.61 (0.08–4.63)	Reference	1.63 (0.89–2.96)	1.90 (0.91–3.96)
**Adjusted OR**	Reference	1.96 (0.93–4.11)*	1.60 (0.64–4.01)*	Reference	**2.41** (1.08–5.38)**	0.69 (0.07–6.47)**	Reference	0.93 (0.48–1.83)***	0.91 (0.38–2.13)***

* Adjusted for institute, female age, fresh/frozen transfer, blastocyst developmental stage, blastocyst age.
** Adjusted for institute, female age, fresh/frozen transfer, blastocyst developmental stage, blastocyst age, TE grade.
*** Adjusted for institute, female age, fresh/frozen transfer, blastocyst developmental stage, blastocyst age, ICM grade.
Live birth was the unit of analysis in this sensitivity analysis.
Bold values refer to statistically significant results.
