# Supplementary material for: Blastocyst quality and reproductive and perinatal outcomes: a multinational multicentre observational study
Source: Hum Reprod. 2023 Oct 24;38(12):2391–9. doi: 10.1093/humrep/dead212 (PMC10694400; doi:10.1093/humrep/dead212)
Supplement: dead212_Supplementary_Table_S4 [file dead212_supplementary_table_s4.pdf]

OXFORD

human reproduction

Human Reproduction, 2023, 1–1
https://doi.org/10.1093/humrep/dead212
Supplementary Data

Supplementary Table S4. Association between different low-grade blastocysts and perinatal outcomes.

Outcomes	Good (N = 1893)	AC (N = 9)	CA (N = 4)	BC (N = 637)	CB (N = 175)	CC (N = 16)
**Preterm birth**	122 (6.4%)	1 (11.1%)	1 (25.0%)	51 (8.0%)	15 (8.6%)	0
**Crude OR**	Reference	/	/	1.26 (0.90–1.77)	1.36 (0.78–2.38)	/
**Adjusted OR**	Reference	/	/	0.97 (0.61–1.53)*	1.09 (0.56–2.10)*	/
**Birthweight Z-score**, **mean (SD)**	0.4 (1.1)	0.4 (0.9)	−0.2 (0.6)	0.6 (1.0)	0.8 (1.0)	0.5 (0.9)
**Coefficient (crude)**	Reference	/	/	**0.27** (0.18–0.37)	**0.40** (0.24–0.56)	/
**Coefficient (adjusted)**	Reference	/	/	0.01 (−0.12 to 0.13)*	0.13 (−0.06 to 0.31)*	/
**Birthweight (VLBW/LBWNBW/HBW)**						
**NBW**	Reference	Reference	Reference	Reference	Reference	Reference
**VLBW**	17 (0.9%)	0	0	4 (0.6%)	1 (0.6%)	0
**Crude OR**	Reference	/	/	0.70 (0.24–2.10)	0.62 (0.08–4.71)	/
**Adjusted OR**	Reference	/	/	0.86 (0.21–3.63)*	1.10 (0.11–10.66)*	/
**LBW**	87 (4.6%)	0	1 (25.0%)	31 (4.9%)	7 (4.0%)	0
**Crude OR**	Reference	/	/	1.07 (0.70–1.62)	0.85 (0.39–1.87)	/
**Adjusted OR**	Reference	/	/	1.03 (0.58–1.82)*	0.78 (0.32–1.93)*	/
**HBW**	31 (1.6%)	0	0	15 (2.4%)	1 (0.6%)	0
**Crude OR**	Reference	/	/	1.45 (0.78–2.70)	0.34 (0.05–2.52)	/
**Adjusted OR**	Reference	/	/	1.32 (0.52–3.37)*	0.19 (0.02–1.68)*	/
**Birthweight (SGA/AGA/LGA)**						
**AGA**	Reference	Reference	Reference	Reference	Reference	Reference
**SGA**	130 (6.9%)	1 (11.1%)	0	22 (3.5%)	4 (2.3%)	0
**Crude OR**	Reference	/		**0.54** (0.34–0.86)	0.38 (0.14–1.04)	/
**Adjusted OR**	Reference	/		1.87 (0.99–3.54)*	1.65 (0.53–5.11)*	/
**LGA**	331 (17.5%)	1 (11.11%)	0	167 (26.2%)	54 (30.9%)	4 (25.0%)
**Crude OR**	Reference	/		**1.61** (1.30–2.00)	**2.00** (1.42–2.82)	/
**Adjusted OR**	Reference	/		1.26 (0.93–1.70)*	1.57 (1.04–2.37)*	/

We did not perform regression analysis for AC, CA, or CC group due to small numbers.
* Adjusted for institute, female age, fresh/frozen transfer, blastocyst developmental stage, blastocyst age, infant gender.
OR, odds ratio; VLBW: very low birth rate; LBW: low birth rate; NBW: normal birth weight; HBW: high birth weight; SGA: small for gestational age; AGA: appropriate for gestational age; LGA: large for gestational age. Results in bold are P<0.05. Crude OR is calculated without any confounders.
Bold values refer to statistically significant results.
