# Supplementary material for: Blastocyst quality and reproductive and perinatal outcomes: a multinational multicentre observational study
Source: Hum Reprod. 2023 Oct 24;38(12):2391–9. doi: 10.1093/humrep/dead212 (PMC10694400; doi:10.1093/humrep/dead212)
Supplement: dead212_Supplementary_Table_S5 [file dead212_supplementary_table_s5.pdf]

OXFORD
human reproduction

Human Reproduction, 2023, 1–1
https://doi.org/10.1093/humrep/dead212
Supplementary Data

Supplementary Table S5. Association between different age blastocysts and reproductive and perinatal outcomes.

Outcomes	D5 low quality embryos (N = 1736)	D6 low quality embryos (N = 1078)	Adjusted OR
**Live birth**	589 (33.9%)	269 (25.0%)	0.58 (0.48–0.70)
**Clinical pregnancy**	778 (44.8%)	370 (34.3%)	0.57 (0.47–0.68)
**Miscarriage**	189 (10.9%)	101 (9.4%)	0.83 (0.63–1.09)
	**D5 low quality embryos (N = 577)**	**D6 low quality embryos (N = 264)**	
**Preterm birth**	47 (8.2%)	21 (8.0%)	1.18 (0.65–2.13)
**Birthweight Z-score**	0.7 (0.5)	0.3 (0.5)	−0.12 (−0.27 to 0.04)
**VLBW**	2 (0.4%)	3 (1.1%)	3.43 (0.41–28.51)
**LBW**	23 (4.0%)	16 (6.1%)	1.71 (0.84–3.48)
**NBW**	541 (93.8%)	240 (90.9%)	Reference
**HBW**	11 (1.9%)	5 (1.9%)	0.75 (0.22–2.59)
**SGA**	17 (3.0%)	10 (3.8%)	1.91 (0.77–4.74)
**AGA**	405 (70.2%)	183 (69.3%)	Reference
**LGA**	155 (26.9%)	71 (26.9%)	1.00 (0.70–1.43)

D5 low quality blastocysts group was the reference group for all comparisons.
Adjusted for institute, female age, fresh/frozen transfer, blastocyst developmental stage; infant gender was adjusted for perinatal outcomes.
